# New Appliances and Things Medical

**Published:** 1895-11-16

**Authors:** 


### NEW APPLIANCES AND THINGS MEDICAL.

[We shall be glad to receive, at our Office, 428, Strand, London, W.C., from the manufacturers, specimens of all new preparations and appliances which may be brought out from time to time.]

### ANGIER'S PETROLEUM EMULSION WITH HYPOPHOSPHITES.

It is only in recent years that petroleum has come to be used in medicine as an internal remedy. This has happened more especially in America, where it is largely produced, and our knowledge of its action is chiefly due to the physicians there. Of late years, owing to improved chemical means, petroleum in several forms has come to be used extensively. More especially is this true in catarrhal diseases. The Angier Company have hit upon the happy idea of emulsifying petroleum and adding hyphophosphites to this preparation. It is well known that when a purified petroleum oil is administered in small doses it has a wonderfully soothing effect on the irritated mucous membranes, not only those with which it comes into contact, but also by absorption those of other regions. The preparation under notice has been used by us clinically for some little time, with very good effects. We find that it is soothing, antiseptic, and slightly laxative. It compares most favourably with cod-liver oil, more especially in adults. Its chief advantage in this respect is its digestibility. There is no nausea present when it is used, nor have we ever noticed diarrhœa, such as is often met with when oily substances are administered to those whose digestion is impaired through the fever of phthisis or the catarrh of a chronic dyspepsia. We can state in conclusion that Angier's preparation is a valuable aid in the treatment of those cases which are mostly of a tedious and prolonged character, aud that it is all the more so inasmuch as it is pleasant to take, being almost tasteless.

### BAXTER'S EXTRA CHOICE OLD SCOTCH WHISKY. (BARLEY-BREE BRAND.)

Many whiskies are, perhaps, better known than the above brand; few, however, deserve more thoroughly the confidence of the medical profession. The distillation of this whisky has been continued for the last hundred years, an experience which has taught the firm all that is to be learnt in the preparation and preservation of a first-class barley spirit. Every bottle of whisky which bears this highly-respected trade mark may be depended upon as of good age and fine flavour, and one that may be safely recommended to the gouty or rheumatic patient. Those who desire to make a trial of this whisky will find their orders promptly attended to by the London agents, The Marza Manufacturing Company, "Ld." 19 and 21, Wilson Street, Finsbury, E.C.

